# A systematic review on the effects of logotherapy and meaning-centered therapy on psychological and existential symptoms in women with breast and gynecological cancer

**DOI:** 10.1007/s00520-025-09519-1

**Published:** 2025-05-10

**Authors:** Andrea Aiello-Puchol, Joaquín García-Alandete

**Affiliations:** 1 University of Valencia, Avda. Blasco Ibáñez, 21, 46010 Valencia, Spain; 2 Department of Personality, Evaluation and Psychological Treatments, University of Valencia, Avda. Blasco Ibáñez, 21, 46010 Valencia, Spain

**Keywords:** Breast cancer, Gynecological cancer, Logotherapy, Meaning-centered therapy, Systematic review

## Abstract

**Purpose:**

Women diagnosed with breast cancer (BC) and gynecological cancer (GC) face psychological and existential challenges common to all cancers, such as anxiety and depression, along with specific issues related to body image and sexuality. Logotherapy and meaning-centered therapy (MCT) have shown positive effects on the psychological well-being of cancer patients.

**Methods:**

A systematic review was conducted following PRISMA guidelines to assess the impact of logotherapy and meaning-centered therapy (MCT) on women diagnosed with BC and GC from January 2014 to December 2024. Empirical research articles published in English were included, while literature reviews, meta-analyses, doctoral theses, preprints, books, and studies involving other cancer types or metastatic/comorbid conditions were excluded. The search was conducted across Web of Science, Scopus, and PubMed databases using terms like uterine cancer, ovarian cancer, breast cancer, mastectomy, logotherapy, and MCT. The Boolean operators AND and OR were used in the Title and Abstract search fields across all three databases, as well as in the Topic search field for the WoS database.

**Results:**

Out of the 36 articles initially obtained, 29 remained after eliminating duplicates and, finally, six papers were selected. The included studies examined various psychological and existential issues in patients with breast and gynecological cancer, including anxiety, distress, depression, hopelessness, death anxiety, post-traumatic stress, perception of physical symptoms, quality of life, post-traumatic growth, spiritual well-being, and meaning in life.

**Conclusion:**

Findings suggest that logotherapy decreases depressive symptoms, anxiety, and post-traumatic stress, whereas improves meaning in life, quality of life, physical symptom perception, and post-traumatic growth in women with BC and GC. Implementing logotherapy in cancer care units through a multidisciplinary approach could be valuable, considering biopsychosocial factors, and incorporating aspects of self-image and sexuality in treatment would also be beneficial.

## Introduction

Cancer affects not only physical health but also psychological and psychosocial well-being, particularly when it disrupts daily life [1]. Gynecological cancers, including cervical, vulvar, endometrial, and ovarian cancers affect women and may progress differently due to variations in their origins, risk factors, and progression [2, 3]. Breast cancer, commonly involving ductal and infiltrating lobular carcinoma, also predominantly affects women [4]. Psychological issues, such as insomnia linked to pain, anxiety, and depression, are common among cancer patients. Depressive symptoms occur in 20–50% of cancer patients, with a higher suicide risk than the general population [5].

### Psychological effects of gynecological and breast cancer

Psychological effects of gynecological and breast cancer include distress, changes in body image, diminished self-esteem, depressive and anxious moods, and post-traumatic stress symptoms [6, 7].

Breast cancer patients experience peak anxiety before mastectomy, with symptoms decreasing over time [8]. Total mastectomy, along with diagnosis, are major sources of distress, leading to more severe depressive symptoms [9]. Cancer treatments and side effects (e.g., mastectomy, alopecia) can negatively impact self-esteem and self-concept, often making patients feel less feminine and sexually attractive, particularly in premenopausal women [10]. However, some patients find meaning in their experience, leading to lower anxiety and depression, higher life satisfaction, and better psychological adjustment [11, 12].

Gynecological cancer patients experience increased anxiety and distress, especially when a recurrence is diagnosed [13]. Physical changes from cancer and its treatment, such as concerns about sexual dysfunction, worsen these symptoms [14]. Anxiety and depression also negatively impact existential well-being and post-traumatic growth [15]. During the diagnosis and treatment of gynecological and breast cancers, in addition to biological problems such as treatment side effects (e.g., hair loss, fertility issues, or pain), psychological and existential problems are particularly significant due to the unique impact these cancers have on femininity, sexuality, and reproductive health. The fear of losing fertility, changes in body image, and concerns about femininity or sexual identity can amplify emotional distress, making these issues more pronounced compared to other types of cancer [16]. The disease and its treatment can affect sexuality and fertility, leading to lower sexual well-being, especially in reproductive-age women, and may result in negative self-image and decreased self-esteem, which, combined with physical changes and financial struggles, reduce quality of life [17, 18]. A comprehensive approach could significantly improve patients’ psychological well-being.

### Logotherapy and meaning-centered therapy in cancer patients

Existential suffering (distress linked to the perception of a threat to one’s existence) [19] has been studied in cancer patients [20]. This suffering manifests in various ways: vulnerability, anxiety, and fear of death; a feeling of losing control over the disease; reduced quality of life; negative emotional reactions; isolation and existential loneliness (feeling alone in facing an unshared fate); a sense of loss, viewing the past as irretrievable and the future as grim; questioning personal dignity; and a deep questioning of life’s meaning [21].

Viktor E. Frankl, the founder of logotherapy, believed that the search for meaning in life is humanity’s primary motivation [22]. According to Frankl, logotherapy is based on three principles: (1) freedom of will: individuals have the freedom to choose their attitudes and responses to life’s circumstances, (2) will to meaning: the primary drive in life is not pleasure (as Sigmund Freud suggested) or power (as Alfred Adler suggested), but the pursuit of meaning, and (3) meaning in life: that is, life has inherent meaning, and each person can find purpose, even in suffering, through discovering meaning in their experiences [22]. Built on these principles, meaning-centered therapy focuses on helping individuals find personal meaning in their lives, especially in the face of suffering, illness, or existential crisis, thereby enhancing psychological well-being and resilience [23].

Meaning in life is linked to perceptions of physical symptoms, life control, mental health, and coping with threats, including in cancer patients [24–26]. Logotherapy helps patients alleviate existential distress, improve mental health, and respond meaningfully to suffering [27]. It has been effective in cancer patients by reducing anxiety, death anxiety, and existential loneliness, among others symptoms, as well as enhancing life meaning [28–30].

Meaning-centered therapy (MCT), based on Frankl’s logotherapy and Irvin D. Yalom’s existential therapy [22, 31], addresses issues like death, freedom, isolation, and meaninglessness. In cancer patients, MCT reduces clinical symptoms, demoralization, and distress, while enhancing hope, purpose, and meaning in life, improving well-being and quality of life, and aiding in better disease coping [32–36]. MCT is more effective than Cognitive Behavioral Therapy (CBT) in increasing meaning in life, with similar outcomes in improving depression and promoting posttraumatic growth at post-treatment and 6-month follow-up [37].

### The present study

The objective of this study is to systematically review existing psychological research to examine how logotherapy and MCT can address the psychological and existential challenges faced by women with gynecological and breast cancer. Specifically, the study aims to identify the aspects of symptomatology (such as emotional distress, anxiety, depression, or existential concerns) in these patients that can be improved through the application of logotherapy and MCT. By reviewing empirical studies, the research seeks to highlight the positive effects of these therapeutic approaches on the mental and emotional well-being of cancer patients.

## Method

### Inclusion and exclusion criteria

The inclusion criteria were: (1) document type: empirical research article published in a scientific journal; (2) language: English; (3) publication period: 2014 to 2024; (4) sample: women with breast and/or gynecological cancer; and (5) design of the study: randomized clinical trial, quasi experimental study, and pre-post study.

The exclusion criteria were the following: (1) document type: literature review, systematic review, meta-analysis, doctoral thesis, preprint, book, or book chapter; (2) sample: patients with other types of cancer, those with metastatic or comorbid breast and/or gynecological cancer.

### Databases and search strategy

The search was carried out between November and December of 2024 across the Web of Science (WoS), Scopus, and PubMed databases. The following terms and their variations were used: uterine cancer, ovarian cancer, ovariotomy, hysterectomy, breast cancer, mastectomy, logotherapy, and meaning-centered psychotherapy. The Boolean operators AND and OR were used in the Title and Abstract search fields across all three databases, as well as in the Topic search field for the WoS database.

The guidelines of the Preferred Reporting Items for Systematic Review and Meta-Analysis (PRISMA) Statement were followed [38]. After identifying and selecting studies, a systematic analysis (study design, diagnosis and stage of the participants’ cancer, intervention applied to the control group, characteristics of the interventions, and effects on symptomathology) was conducted. The screening, selection, and analysis of the documents were managed by all authors of the present study.

In this study, a snowball search was not performed because the search strategy in electronic databases already exhaustively covered the relevant literature available. The search focused on recognized databases specific to the research area, which ensured the inclusion of key studies. Despite not performing this strategy, the studies identified in the databases were representative and complete for the objectives of the review.

## Results

### Document selection process

**Fig. 1:**
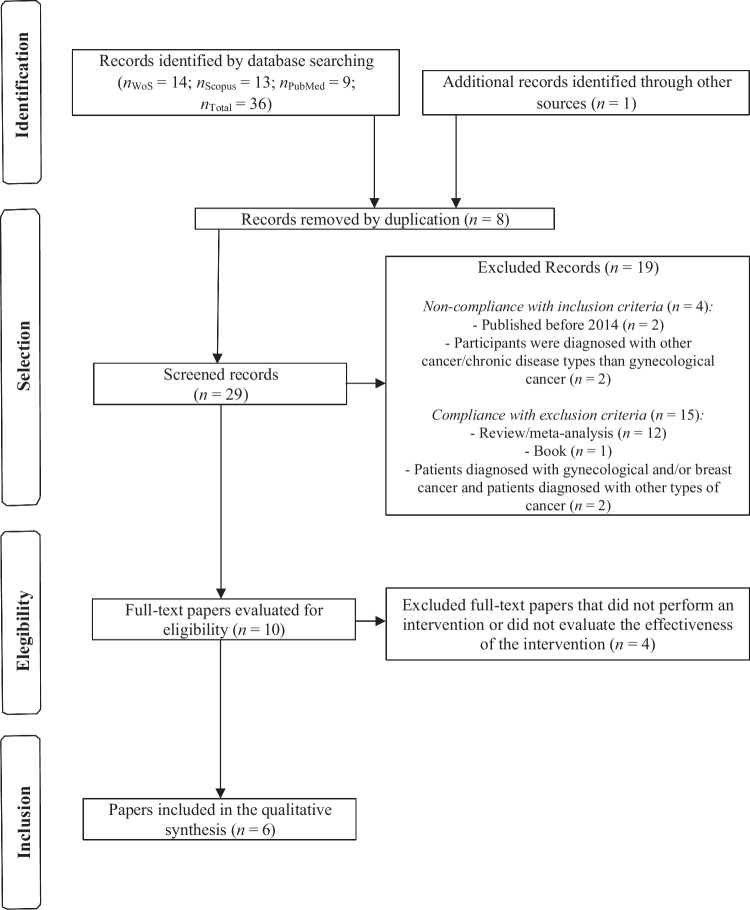
PRISMA flowchart

Initially, 36 articles were obtained in the databases used (*n*_WoS_ = 14, *n*_Scopus_ = 13, *n*_PubMed_ = 9) and one article in the references of one of the obtained works, which were reduced to 29 after eliminating the duplicates. Finally, six papers were selected [39–44] (Fig. 1), whose characteristics and main results are shown in Table 1.

**Table 1:** Main data from the studies reviewed in this work

Authors	Year of publication	Country of publication	Objective	Study design	Participants	Intervention	Sessions	Main results and conclusions
Alreda et al. [39]	2022	Iraq	To investigate the effectiveness of group logotherapy on life expectancy and death anxiety in women with BC	Quasi-experimental study with a pre-test, post-test, and control group design	40 women with BC (control group, *n* = 20; experimental group, *n* = 20)	Experimental condition: logotherapy Control condition: no intervention	The logotherapy training was performed in 10 sessions of 2 h and once a week based on the protocol of logotherapy training	Significant difference between post-test of groups in life expectancy and death anxiety Logotherapy decreased death anxiety and raised life expectancy in women with BC, and as a psychological intervention can improve the quality of life of people with chronic diseases such as BC
Aydin & Kabukcuoğlu [40]	2023	Turkey	To examine the effects of logotherapy-based, nurse-guided meaning attribution conversations on traumatic stress symptoms, post-traumatic growth, spiritual well-being, and life meaning attribution in gynecological cancer patients	Single-blind, randomized controlled trial	68 women with GC (control group, *n* = 34; experimental group, *n* = 34)	Experimental condition: conversations to attribute meaning (based on logotherapy) guided by nursing Control condition: usual nursing care	Women in the intervention group underwent seven meaning attribution conversations sessions Women in the control group only received routine nursing care	Significant differences in the post-intervention and follow-up mean TSSS, PTGI, and MLQ scores between groups Mean total SWBS scores were not significantly different between groups Meaning attribution conversations decreased the traumatic stress symptoms and increased the post-traumatic growth and ability to attribute meaning to life, but did not affect the spiritual well-being
Mohabbat-Bahar et al. [41]	2014	Iran	To investigate the effectiveness of group logotherapy on the reduction of anxiety in women with BC	Quasi-experimental with pre-test, post-test and control group	30 women with BC (control group, *n* = 15; experimental group, *n* = 15)	Experimental condition: logotherapy Control condition: no intervention	The experimental group received Logotherapy-based group counseling for eight sessions	Group logotherapy was significantly effective in reducing anxiety in women with BC Psychosocial interventions can be used to reduce anxiety in the BC patients
Raji et al. [42]	2022	Iran	To determine the effects of logotherapy along with nutrition counseling on psychological status, QoL, and dietary intake among BCSs who were diagnosed with depression	Randomized Clinical Trial	90 women with BC who had already completed treatment (control group, *n* = 44; experimental group, *n* = 46)	Experimental condition: logotherapy + nutritional counseling Control condition: nutritional counseling	The logotherapy was performed weekly for two months as a group therapy	All dimensions of QoL, anthropometric measurements, and the CES improved significantly in both groups after 8 weeks A combination of nutrition counseling and logotherapy resulted in a significant reduction in anxiety and depression scores compared with the nutrition counseling alone In addition, participants who received logotherapy plus nutrition counseling significantly consumed less energy, carbohydrate, and fat intake after 8 weeks compared with the control group Logotherapy + nutrition education would be an important step in improving anxiety, depression, and QoL of patients with BC who had depressive symptoms
Soetrisno et al. [43]	2017	Indonesia	To analyze the effect of logotherapy on the expression of cortisol, HSP70, and the Beck Depression Inventory and to conduct pain assessments in advanced cervical cancer patients	Pretest–posttest control-group	30 women with advanced CC (control group, *n* = 15; experimental group, *n* = 15)	Experimental condition: logotherapy Control condition: usual treatment	Six meetings of 45-min sessions each week in addition to the standard treatment Patients in the control group only receive the standard treatment	Significant difference between groups after the treatment, except for on HSP70 Logotherapy affects the expression of cortisol, BDI, and pain scales in advanced cervical cancer patients, and it does not affect the expression of HSP70
Sun et al. [44]	2021	Taiwan	To evaluate the effects of logotherapy on distress, depression, and demoralization in breast cancer and gynecological cancer patients	Quasi-experimental	61 women with GC and BC cancer (control group, *n* = 30; experimental group, *n* = 31)	Experimental condition: logotherapy + nutrition counseling Control condition: psychoeducation about the necessary care for BC and GC	Participants in the experimental group received logotherapy four to six times during the 12 weeks of intervention	A combination of logotherapy + nutrition counseling resulted in a significant reduction in anxiety and depression scores compared with the nutrition counseling alone Participants who received logotherapy + nutrition counseling significantly consumed less energy, carbohydrate, and fat intake after 8 weeks compared with the control group Logotherapy + nutrition education would be an important step in improving anxiety, depression, and QoL of patients with BC who had depressive symptoms

*BC;* breast cancer, *CC;* cervical cancer, *GC;* gynecological cancer, *HPS70;* 70 Kilodalton Heat Shock Proteins, *QoL;* Quality of Life as measured through the EORTC QLQ-C30 and QLQ-BR23 questionnaires, *CES;* Compulsive Eating Scale, *TSSS;* Traumatic Stress Symptom Scale, *PTGI;* Post-Traumatic Growth Inventory, *MLQ;* Meaning of Life Questionnaire, *SWBS;* Spiritual Well-Being Scale

### Systematic analysis

#### Study design

Two studies conducted randomized clinical trials [40, 42], while four others employed a pre-post design with a control group [39, 41, 43, 44]. Of these last works, three of them used a quasi-experimental design [39, 41, 44]. Two studies specified the method of assignment to experimental conditions, with both using simple randomization [39, 43].

#### Diagnosis and stage of the participants’ cancer

The studies reviewed analysed the effects of logotherapy in women with breast cancer alone [39, 41, 42], endometrial, uterus, and ovarian cancer [40], ovarian cancer [43], and breast, ovarian, cervical, and endometrial cancer [44].

Participants had stage IIB-IV cancer [43], stage II breast or gynecological cancer (47.2%), with a notable percentage of cases in stage III or higher (36.1%) [44], mainly stage II breast cancer (41.3%) in the experimental group while most had suffered from stage I breast cancer (43.2%) in the control group [42], and stage II and III gynecological cancer in similar proportion in both the control and experimental groups [40].

#### Intervention applied to the control group

In three studies, the control group did not receive any intervention [39, 41, 43]; in one study, the control group received usual nursing care [40]; in one study, the control group received psychoeducation on the care needed for gynecological and breast cancer [44], and in another study, the control group received nutritional counseling [42].

#### Characteristics of the interventions

Five studies applied a strictly logotherapy intervention in the experimental group [39, 41–44]. In three of them, the intervention was group, although the number of participants in the groups was not specified [39, 41, 42].

Mohabbat-Bahar et al. [41] carried out logotherapy adapted to Iranian and Islamic culture. These authors defined the goals and briefly summarized the actions carried out in each session. The main topics addressed were concept of meaning in life, freedom of will, spirituality, the sources of meaning described by Frankl, and cancer as a source of meaning. The techniques used were paradoxical intention and reflection for the modification of attitudes and, at the end of the intervention, each patient proposed new life goals.

Soetrisno et al. [43] provided a brief summary of each session, without specifying the objectives or techniques used. Some of the aspects worked on in the intervention were the concepts of “freedom of will” and “will of meaning”, meaning in life and sources of meaning. There were also moments of treatment dedicated to emotional ventilation, the development of coping strategies to cope with situations associated with the disease, and self-knowledge and self-confidence. Religion and spirituality were used to foster acceptance of cancer and the search for meaning in the experience.

Sun et al. [44] briefly described the general lines of the logotherapeutic intervention, which consisted of a review of the life history, detecting the most relevant events and those that gave meaning to it. Likewise, the sources of meaning were shown, trying to make the patient find a meaning to suffering.

Alreda et al. [39] did not describe in detail the group logotherapy intervention carried out, limiting to reporting the number of sessions and the key concepts covered in each session. During the therapeutic sessions, the sources of meaning in life, freedom of will, the will to meaning and the meaning of suffering and death were worked on. In addition, religiosity was included as a therapeutic resource.

Raji et al. [42] combined two types of interventions in the experimental group. On the one hand, nutritional counseling (four sessions), where the main types of food, the components of a balanced diet (also addressing practical issues such as buying food), the relationship between mood and food and coping with the consequences of cancer that affect food were shown. On the other hand, in group logotherapy, concepts such as ‘freedom of will’, spirituality and impermanence of the human being were worked on, and the different sources of meaning and techniques of logotherapy (hyperreflection, paradoxical intention, reflection) were shown in order to find meaning in life and stressful life events (among these, cancer). Likewise, goals and future plans were established according to the personal values of each participant. The description of nutritional counseling was made in greater detail than the description of the logotherapeutic intervention.

Aydin and Kabukcuoğlu [40] based the intervention on logotherapy, specifically conversations were held in order to make sense of the experience, with individual interventions. These authors discussed the main concepts of logotherapy (freedom of will, will to meaning, and meaning in life), the meaning attributed to cancer so far, life history, and how it had influenced meaning in life. Different sources of meaning were also exposed, motivating patients to identify aspects of their lives that fit into them. They also specified the topics, techniques and objectives of each session.

#### Effects on anxiety symptoms

Two studies analyzed the direct effect of logotherapy on anxiety. Mohabbat-Bahar et al. [41] found statistically significant differences between the logotherapy vs. no intervention groups. Raji et al. [42] analyzed the effects of logotherapy together with nutritional counseling (compared to receiving nutritional counseling alone), without finding significant differences between the groups.

Soetrisno et al. [43] studied anxiety levels indirectly, comparing the levels of cortisol and 70 Kilodalton Heat Shock Proteins (HSP70) in the blood of a group that received logotherapy compared to one that received the usual treatment. Only significant differences in cortisol levels were found. These authors hypothesized that the non-significance of the differences in HSP70 was due to the method used to extract the sample.

Sun et al. [44] compared the effects of logotherapy and psychoeducation on care for gynecologic and breast cancer on distress, without finding statistically significant differences between the groups.

Alreda et al. [39] found significant differences in death anxiety levels between the logotherapy and no intervention groups, with a large effect size.

#### Effects on symptoms of traumatic stress

Aydin and Kabukcuoğlu [40] compared the effects of logotherapy-based intervention and usual nursing care, finding statistically significant differences between groups (with a large effect size) that were maintained one month after the intervention.

#### Effects on depressive symptoms and variables associated with depression

Three studies found positive effects of logotherapy in reducing depressive symptomatology. Using the BDI, significant differences were found in the groups of logotherapy and nutritional counseling vs. nutritional counseling [42] and logotherapy vs. usual treatment [43]. Significant differences were also found between the logotherapy vs. psychoeducation groups on the care needed for gynecologic and breast cancer [44].

The other studies found significant differences in perceived life expectancy between the logotherapy and absence of intervention groups [39], significant differences with a large effect size, which were sustained at a one-month follow-up after the intervention concluded [40], no significant differences between logotherapy accompanied by nutritional counseling compared to receiving nutritional counseling alone in binge eating [42], and significant differences in the total scale scores and in the subscales of loss of meaning, dysphoria, discouragement, helplessness, sense of failure, and discouragement [44].

#### Effects on quality of life and perception of physical symptoms

One study found statistically significant differences between the groups of logotherapy and nutritional counseling vs nutritional counseling in quality of life (specifically in the physical, role, emotional, and social areas) [42].

Regarding the outcomes of logotherapy on the perception of physical symptoms, in one study, the results revealed significant differences in the perception of physical symptoms in women who received logotherapy treatment together with nutritional counseling vs. those that received nutritional counseling in pain, fatigue, loss of appetite, and diarrhea [42], and in another study in which the effects of logotherapy compared to usual treatment were analyzed, significant differences between groups were found [43].

#### Effects on spiritual well-being

Only the study by Aydin and Kabukcuoğlu compared the effects of logotherapy-based intervention and usual care on spiritual well-being, finding a non-significant difference [40].

#### Effects on post-traumatic growth

Aydin and Kabukcuoğlu found that the logotherapy-based intervention group showed a significant higher level of post-traumatic growth than the usual care group [40].

## Discussion

The aim of this study was to analyze the effects of logotherapy and MCT on the symptomatology of breast and gynecological cancer. To this end, a systematic review of six articles was carried out [39–44], and selected following the PRISMA method.

The results of the studies reviewed show that logotherapy has a positive effect on the symptoms of anxiety, death anxiety, and post-traumatic stress in patients with breast and gynecological cancer [40, 41, 43], although no significant differences in anxiety or distress were detected in two studies [42, 44]. Likewise, a significant reduction in depressive symptoms and hopelessness was described, accompanied by an increase in meaning in life [39, 40, 42–44]. In addition, an improvement in the perception of physical symptoms and quality of life was found [42, 43], as well as increased spiritual well-being and post-traumatic growth [40].

Certain circumstances associated with the medical intervention process, such as mastectomy in breast cancer patients and the diagnosis of recurrence in the case of gynecological cancer patients, have a significant psychological impact [13, 45]. The studies by Alreda et al. and Mohabbat-Bahar et al. included breast cancer patients who had already undergone mastectomy and who had been diagnosed for more than six months, respectively, finding positive effects of logotherapy on hopelessness [39] and anxiety symptoms [41]. Sun et al., who included women with non-recurrence, gynecologic and breast cancer, found that logotherapy produced significant effects in reducing depressive symptoms, but not in distress [44].

These results are similar to those found in previous studies that evaluated the effects of the application of logotherapy and MCT to patients regardless of the type of cancer, in relation to anxiety (in general and in the face of death specifically) [28, 29, 32, 35, 44, 47], depressive symptoms [48] and quality of life and perception of physical symptoms [32, 33].

The findings of Aydin and Kabukcuoğlu [40] regarding post-traumatic growth differ from those found in other studies, which found no significant effects of logotherapeutic intervention on this variable [49, 50].

## Limitations of the reviewed studies

Most of the studies reviewed in the present work were pre-test/post-test or quasi-experimental. These designs are useful but have some limitations that can introduce biases, which can undermine conclusions about cause and effect and affect the validity of results. Pre-test post-test designs may fail to account for external factors, while quasi-experimental designs risk selection bias due to the lack of randomization. Future research should prioritize randomized controlled trials (RCTs) with larger sample sizes, longer follow-up periods, and clear inclusion/exclusion criteria to control bias. RCTs enhance internal validity and help establish cause-and-effect relationships. By addressing these issues, more reliable and generalizable findings can be achieved, improving real-world applicability.

Likewise, none of the studies reviewed in the present work showed that the intervention had positive effects on body image and on the reduction of sexual alterations, areas affected in these patients [52]. Other interventions, such as individual or group CBT and psychoeducational sessions have shown positive effects in these areas [53, 54].

The studies reviewed in this work also did not analyze the effect of logotherapy on certain relationships found in the literature, such as the relationship between meaning in life and psychological adjustment to disease and functioning in relation to health in women with breast cancer [12]. Nor did Aydin and Kabukcuoğlu [40] analyzed in their work (whose participants suffered from gynecological cancer and had received chemotherapy treatment) whether logotherapy reduced the distress and anxiety associated with the changes caused by such treatment [14]; these authors also did not assess whether changes in post-traumatic growth had had positive effects on anxious and depressive symptoms, variables that maintain a negative relationship with each other [15]. Another aspect that was not evaluated in the interventions was the appropriateness of the intervention before patients underwent mastectomy or before the diagnosis of recurrence.

Another limitation of the reviewed studies was the lack of representation of European, American or African countries: two interventions were carried out in the Middle East (Iraq and Iran), one in Turkey [40], one in Indonesia [43] and one in Taiwan [44], which may affect the generalizability of the results obtained. This limitation was also pointed out in a systematic review and meta-analysis on interventions for death anxiety in patients with chronic diseases (cancer, among others) [46]. The high prevalence of studies on logotherapy in these countries may be due to the fact that in the Middle East, Turkey and Indonesia, research is more interested in interventions that address spirituality in the care of patients with physical illnesses [43], as is the case of logotherapy [51], as these countries attach more importance to religion compared to European countries [55]. Therefore, in view of the positive results obtained in previous research on MCT in cancer patients in Europe [49, 50], it would be appropriate to carry out research in Europe with the aim of testing whether the results found can be generalised to European women with gynecological and breast cancer.

Finally, since the reviewed studies were written in English, there is an inherent bias toward research from English-speaking countries or those with a high proficiency in the language. This can lead to an underrepresentation of studies from non-English-speaking regions or researchers, potentially skewing the overall findings or conclusions.

## Limitations of the present study

This study has some limitations that should be taken into account. First, it was not registered with a clinical trial or systematic review database, which may affect the transparency and reproducibility of the review process. Therefore, the absence of a registration may limit the ability to track any potential updates or amendments to the review protocol.

Second, the assessment of risk of bias was not included due to the nature of the studies reviewed. Many of the included studies were either non-randomized or had methodological limitations that made it challenging to apply a standardized risk of bias tool across all studies. Additionally, the studies varied in design, population, and intervention details, making it difficult to consistently evaluate bias risks. Despite this, efforts were made to ensure the studies selected were relevant and methodologically sound based on the inclusion criteria. Future reviews may incorporate a more detailed risk of bias assessment as the field evolves and more rigorous studies are conducted.

Third, the focusing on the logotherapeutic interventions rather than conducting an in-depth exploration of specific psychological or existential problems, may limit the understanding of the underlying issues that these therapies aim to address. By not thoroughly investigating the specific psychological and existential challenges faced by patients, the present review may not capture the full range of emotional, cognitive, and existential difficulties experienced by the target population. This lack of depth could affect the interpretation of how effective the interventions are in addressing these complex issues and may overlook important factors that contribute to the success or limitations of the therapies. An in-depth exploration of the psychological and existential problems could provide a more nuanced understanding of the interventions’ impact and their potential to alleviate these issues.

## Clinical implications

The reviewed studies in the present work examined the effects of logotherapy on women with various cancers, including breast, ovarian, endometrial, and gynecological cancers. Participants were primarily in stages II–III of cancer, with the experimental group receiving logotherapy, while control groups varied in their interventions, including no intervention, usual nursing care, psychoeducation, or nutritional counseling. Interventions focused on concepts such as meaning in life, freedom of will, spirituality, and the sources of meaning, often involving group therapy or individual sessions. The results of the reviewed studies showed significant positive effects of logotherapy on reducing anxiety, depressive symptoms, traumatic stress, and improving quality of life, although some studies did not find significant differences in all outcomes. Logotherapy also positively impacted post-traumatic growth, while effects on spiritual well-being were less clear.

Given the positive effects of logotherapy in breast and/or gynecological cancer patients, as well as the approach by Aydin and Kabukcuoğlu where nurses implemented the intervention, it would be beneficial to incorporate logotherapy into cancer patient units [40]. This could be used as a therapeutic tool, as suggested by Frankl [22], by both psychologists and other healthcare professionals in their patient interactions. A multidisciplinary approach would also help determine the best timing for these interventions, considering biopsychosocial factors.

A limitation of the logotherapy interventions in the reviewed studies was their failure to address changes in body image and sexuality in breast and/or gynecological cancer patients. Integrating logotherapy with other therapies could offer a more holistic approach, allowing for the assessment of both therapies’ specific positive effects, as well as their interaction [56–58]. This could improve life meaning, self-image, and sexual functioning, while reducing existential suffering, physical symptoms (e.g., fatigue, nausea), and psychological issues (e.g., depression, anxiety, traumatic stress) [59].

## Data Availability

No datasets were generated or analysed during the current study.
